# Behavior and physiology in female *Cricetulus barabensis* are associated with the expression of circadian genes

**DOI:** 10.3389/fendo.2023.1281617

**Published:** 2024-01-04

**Authors:** Hanyi Zhu, Ming Wu, Junjie Mou, Xueqi Yang, Qian Xu, Yongjian Zhang, Hao Zhang, Xinran Wang, Huiliang Xue, Jinhui Xu, Lei Chen, Laixiang Xu

**Affiliations:** College of Life Sciences, Qufu Normal University, Qufu, China

**Keywords:** *Cricetulus barabensis*, circadian genes, behavior, physiology, daily rhythm

## Abstract

The circadian clock regulates the behavior, physiology, and metabolism of mammals, and these characteristics, such as sleep-wake cycles, exercise capacity, and hormone levels, exhibit circadian rhythms. Light signaling is the main stimulator of the mammalian circadian system. The photoperiod regulates the reproductive cycle of seasonal breeding animals, and the circadian clock plays a pivotal role in this process. However, the role of the clock in coordinating animal behavior and physiology in response to photoperiodic changes needs further investigation. The present study investigated the changes and correlation of behavioral activities, physiological indicators, and gene expression in female striped hamsters (*Cricetulus barabensis*) within 24 h under a 12L:12D photoperiod. We found that the daily rhythms of sleep-wake and open field were significant in hamsters. The expression of clock genes, melatonin receptor genes, and genes involved in general metabolism oscillated significantly in central and peripheral tissues (brain, hypothalamus, liver, ovary, and thymus) and was significantly associated with behavior and physiology. Our results revealed that the neuroendocrine system regulated the rhythmicity of behavior and physiology, and central and peripheral clock genes (*Bmal1*, *Clock*, *Per1*, *Per2*, *Cry1*, and *Cry2*), melatonin receptor genes (*MT1*, *MT2*, and *GPR50*), and metabolizing genes (*SIRT1*, *FGF21*, and *PPARα*) played important roles. Our results suggest that central and peripheral circadian clocks, melatonin receptors, and genes involved in general metabolism may play key roles in maintaining circadian behavior and metabolic homeostasis in striped hamsters. Our results may have important implication for rodent pest control.

## Introduction

Circadian rhythm refers to the 24 h oscillation pattern of biological processes (1). A core oscillator that exhibits daily rhythm regulates behavior (e.g., sleep-wake) (2), physiology (e.g., metabolism and hormones) (3–5), and the expression of genes (e.g., clock genes) (6) in mammals. The core oscillator endogenously maintains the daily rhythms of the animal by receiving input signals, including photoperiods, and changing to output signals (7). The core oscillator is primarily located in the suprachiasmatic nucleus (SCN) of mammals and consists of a transcriptional/translational feedback loop formed by a set of clock genes (*Bmal*1, *Clock*, *Pers*, and *Crys*) (8). BMAL1 and CLOCK proteins form a dimer complex that binds to *E*-box promoter regions (CACGTG) of *Pers* (*Per1*, *Per2*, and *Per3*) and *Crys* (*Cry1* and *Cry2*), which play positive regulatory roles in the core loop (9–11). Conversely, PER and CRY proteins are phosphorylated by casein kinase 1ε (12) and transferred from the cytoplasm to the nucleus to inhibit the pro-transcriptional activity of the CLOCK/BMAL1 protein dimer complex and act as a negative regulator in the core loop. This interlocked transcriptional/translational feedback loop is approximately 24 h and thereby reduces the pro-transcriptional effect of the entire circadian clock system to a low level (13).

In addition, mRNA products of clock genes are also found in peripheral organs, including the liver, ovary, and thymus (7, 14, 15). It was generally believed that the synchronization of clock genes in peripheral tissues to light signals (light-dark cycle) was primarily generated by the SCN (16). However, recent studies have shown that, in addition to light signals, such as rhythmically feeding schedule, can directly reset peripheral clocks (17, 18). In addition, the molecular mechanisms of clock genes located in central and peripheral tissues were generally similar (19, 20), but differences between tissues were noted. While the autonomy of peripheral clocks has not been fully elucidated, there are several evidence found that the peak phase of clock genes differed in peripheral tissues, and the molecular response of circadian clocks appeared to be tissue-dependent (15); *Bmal1* mRNA expression was increased in peripheral tissues in clock mutant mice, and while the rhythm was lost in the SCN (21); The amplitudes of renal clock gene amplitudes were strongest and testicular clock genes were weakest as compared to other peripheral organs in hamsters (7); High-throughput transcriptomics and metabolomics analyses to demonstrate that the liver retained an intrinsic clock function and some autonomy in Bmal1-null (whole-body knockout) mice, even in the absence of rhythmic oscillation in other tissues (22). Moreover, SCN-ablation experiments revealed that the SCN was positioned at the top of the hierarchy in mammals and controlled the peripheral clocks by influencing the concentration of multiple neurohumoral factors (23, 24). In addition, the autonomy of peripheral tissues has not been fully elucidated.

Melatonin (MT) is a neurohormone secreted by the pineal gland at night, and it has long been recognized as the main endocrine output signal of the endogenous circadian clock system (25), which synchronizes peripheral tissues with the circadian clock. MT has a high affinity for two melatonin receptors (MT1 and MT2) in mammals. The orphan receptor (G protein-coupled receptor 50, GPR50) may also regulate MT signaling. Although this receptor does not bind MT, it forms a heterodimer with MT1 to inhibit its activity (26). There is also an interaction between energy metabolism and the circadian clock (27, 28), and genes involved in metabolism are rhythmically expressed (29, 30). Sirtuin 1 (SIRT1) is an NAD+-dependent histone deacetylase that is involved in the caloric restriction response in mammals (31). SIRT1 was recently identified as a novel regulator of circadian genes (32, 33) by directly acting on peroxisome proliferator-activated receptor alpha (PPARα) to activate the expression of genes involved in lipolysis (34). PPARα regulates the expression of fibroblast growth factor 21 (FGF21) and affects its daily rhythm (35). Although core mammalian clock genes have been clearly defined, the mechanisms of central and peripheral clock genes in maintaining metabolic homeostasis and energy balance are not clear (36).

The sleep-wake cycle is the most common circadian rhythm in animals (37), and it is caused by a variety of brain structures and neurotransmitter systems that exhibit strict rhythmicity. The circadian rhythm of the sleep-wake cycle is closely linked to the pacemaker, and this coordinated neural activity drives alternating patterns of behavior, including rest, activity, body posture, and changes in response to stimuli (38). The SCN has been shown to be the primary pacemaker in regulating the sleep-wake cycle. However, the specific roles of circadian clocks in multiple brain regions and peripheral organs in controlling sleep remains unclear (39).

Most seasonal breeding animals living in temperate or boreal regions adjust their physiology, behavior, and morphology in response to changes in the environment and synchronize with the seasons (i.e., photoperiod) (40). Circadian clocks are involved in the photoperiod regulation of seasonal breeding in animals (41). Studying the role of circadian clocks in seasonal breeding may elucidate on the mechanisms of clocks regulation of seasonal physiological and behavioral changes.

The specific patterns of circadian rhythms that exist in animals is closely related to its ecological niche (42). The striped hamsters are widely distributed in grasslands and farmlands and are the prey of a variety of predators (such as *Athene noctua*) (43). These hamsters have a wide distribution, high reproductive capacity (a litter of four to nine offspring) (44), and typical daily rhythm characteristics. These animals participate in many ecological processes and are important species in maintaining grassland ecosystems and biodiversity. They feed primarily on crop seeds (e.g., soybeans, mung beans, and wheat), grass seeds, and beetles. The composition of their diet changes depending on the region and environment. Inappropriate daily activity cycles increase the risk of predation by predators. Xu et al. found that, a gene regulating reproduction, *Kiss-1* (which encodes Kisspeptin-1), was expressed in several tissues (the hypothalamus, ovary, and testis) of hamsters in tissue- and sex-dependent manner, and showed different expression trends with seasonal changes (45). A novel hypothalamic-pituitary RFamide-related peptide (RFRP), identified in quail in 2000, is a homolog of the avian gonadotropin-inhibitory hormone (GnIH) and inhibits gonadotropin release by acting directly on the pituitary (46). In striped hamsters, RFRP-3 has the highest expression in the hypothalamus of breeding males and the lowest expression in the hypothalamus of breeding females, which suggests that the regulation of reproduction by RFRP-3 depends on sex and developmental state (47). Aggressive behaviors, resting metabolic rate (RMR), and plasma concentrations of estradiol in female hamsters are different across the estrous cycles. Aggressive behavior of the hamsters in estrus decreased, and the estradiol in plasma and RMR levels increased when facing a male hamster (48).

The present study, obtained a spatiotemporal map of genes, metabolism, and behavior by analyzing the 24 h rhythmicity of six circadian clock genes (*Bmal1*, *Clock*, *Per1*, *Per2*, *Cry1*, and *Cry2*), three melatonin receptor genes (*MT1*, *MT2*, and *GPR50*), and three genes involved in general metabolism (*SIRT1*, *FGF21*, and *PPAR*α) in the central (brain and hypothalamus) and peripheral tissues (liver, ovary, and thymus), three physiological indices (serum melatonin levels, blood sugar levels, and resting metabolic rate), and three behavioral indices (sleep-wake, open field, and elevated plus maze) of the striped hamster. It is worth noting that elevated plus maze is a behavioral test to detect the aversion of rodents to open and elevated areas and the response to novel environment (49). This behavioral test was used to assess the activity of hamsters and their responses to new environments at different time points in this study. We established the relationships of genes, metabolism, and behavior in time and space, and revealed the mechanisms of coordination and communication between these factors. By exploring the biological correlation of circadian rhythm of striped hamster, it is helpful to find its active time, providing theoretical basis for field trapping to control rodent infestation in farmland, and clarifying its possible role in the ecological niche.

## Materials and methods

### Experimental animals

The striped hamsters were captured in March of the year using the same manner as our previous study (47). Hamsters were brought to the laboratory of Qufu Normal University, housed individually in plastic cages (32 cm × 21 cm ×16 cm) and maintained under a light/dark cycle of 12 hours (12L:12D) (illumination time, 08:00-20:00; light intensity, 300 lx) at a temperature of 22 ± 2°C with free access to water and food. Thirty-six female hamsters (20-30 g, 6 months of age) were selected for this experiment and acclimated under a photoperiod of 12L:12D for 8 weeks. The Ethics Committee of Qufu Normal University approved all procedures and surgeries.

### Estrous cycle staging identification

Vaginal cells were collected using aseptic vaginal lavage (50). Briefly, the opening of the vaginal canal was washed with sterile water, and sterile water was used at the opening of the vagina canal to aspirate the vaginal cells by gentle pipetting several times. Vaginal cells were spread on sterile glass slides, fixed for 10-15 s, and air-dried.

Slides with vaginal cells were stained with 0.1% crystal violet (Aladdin, China) stain for 1 min, then transferred in ddH_2_O to clean slides for 1 min and this process was repeated. Excess ddH_2_O was aspirated from the edge of the slide using filter paper, and the cells were covered with a coverslip. Approximately 15 μL of glycerol was placed on top of the slides and coverslips and allowed to dry at room temperature. The types of vaginal cells were observed under a light microscope (**Supplementary Figure S1**). During proestrus, cell type is almost exclusively round and well-structured nucleated epithelial cells (**Supplementary Figure S1D**). During estrus, cornified epithelial cells mainly appear in clusters in the smear, and nucleated epithelial cells are occasionally seen (**Supplementary Figure S1E**). During metestrus, cell types are mainly leucocytes and cornified epithelial cells (**Supplementary Figure S1F**). During diestrus, leukocytes accounts for the majority of the smear, with occasional nucleated epithelial cells and cornified epithelial cells (**Supplementary Figure S1G**). To eliminate the disturbance of the estrous cycle in female hamsters, we performed all experiments when hamsters were in diestrus.

### Behavioral tests

Animals were acclimated to the environment at 12L:12D for 8 weeks, and the sleep-wake behavior test was performed. After the animals rested for three days, the open field behavior was measured every 4 hours throughout the day. After the animals rested for three additional days, elevated plus maze behavior was measured at 4 h intervals throughout the day. To avoid continuous behavioral tests that may interfere with the accuracy of animal behaviors, such as adaptation, we performed subsequent behavioral tests after detecting the same behavior of all animals. The first animal tested was randomized in all behavioral tests. All hamsters were unequally exposed to behavioral assays at ZT0, and the time point of the first exposure was randomized.

#### Sleep-wake cycle

Sleep-wake cycle was measured for 1 day after the hamster was moved into new chambers and adapted to it for 1 h. The circadian behavioral characteristics of hamsters were recorded for 24 h using the IR Actimeter (Panlab, Harvard Apparatus, Spain). Hamsters were considered sleeping when they were motionless for at least 40 s (51, 52). The sleep duration was counted every 30 min, for a total of 48 counts.

#### Open field

At six zeitgeber time points [ZT; ZT0 (08:00), ZT4, ZT8, ZT12, ZT16, and ZT20] on the same day, hamsters were placed into the IR Actimeter for 1 min to acclimate, and then their behavioral indicators were observed and recorded for 10 min. The field (50 cm × 50 cm) was divided into an inner zone (30 cm × 30 cm) and an outer zone using the ACTITRACK program (Panlab, Harvard Apparatus, Spain). After the test, the open field was wiped with 75% alcohol to avoid the influence of residual urine and feces on the hamsters for the next experiment. The room where behaviors were tested at night was illuminated with red LED lights (637 nm).

#### Elevated plus maze

The elevated plus maze experimental apparatus consisted of two open arms and two closed arms. At the six time points of ZT0, ZT4, ZT8, ZT12, ZT16, and ZT20, the behavioral indices of hamsters in the elevated plus maze were tested within 5 min. The room where behaviors were tested at night was illuminated with red LED lights (637 nm).

### Measurement of resting metabolic rate

The resting metabolic rate was measured using an open-flow respirometry system (Q-Box RP2LP, Qubit, Canada). The system had two analyzers, a CO_2_ infrared gas analyzer (IRGA; 0-10%) and an O_2_ infrared analyzer (IRGA; 0-100%), which measured CO_2_ and O_2_ in the breathing chamber, respectively. At ZT0, ZT4, ZT8, ZT12, ZT16, and ZT20, the stable oxygen consumption was read after the hamsters were placed in the apparatus for 1 h, and the results of at least three measurements were averaged.

### Sample collection

After acclimation, hamsters (n = 36) with similar body weights were divided into 6 groups (n = 6 per group), and sacrificed via CO_2_ asphyxiation at ZT0, ZT4, ZT8, ZT12, ZT16, and ZT20. Blood samples were immediately collected from the heart within 2 min, and blood glucose levels were assessed using a blood glucose monitor (Yuwell 580, Jiangsu, China). The remaining blood samples were incubated at 4°C for 30 min, followed by centrifugation at 3000 rpm for 15 min at 4°C. The supernatant was carefully absorbed, and the serum was stored at -80°C for further analyses. The hypothalamus, brain (i.e., cerebral cortex), liver, ovary, and thymus were removed and immediately flash-frozen in liquid nitrogen and stored at -80°C for further analysis.

### Measurement of serum MT concentration

An enzyme-linked immunosorbent assay (ELISA) from Labsystems Multiskan MS 352 (Shanghai Hengyuan Biological Technology Cat# HS022-Hr, RRID: AB_2924940) was used to measure serum MT concentration (44). The detection range of the MT kit was 15-600 pg/mL, the minimum detected concentration was 3.75 pg/mL, and the intra- and inter-assay variations of the MT kit were 5.4% and 7.2%, respectively. Each serum sample was run in duplicates.

Blank wells, standard wells and serum sample (all samples of day and night) wells were arranged in a 96-well plate. Fifty microliters of standard sample and 50 μL of serum sample were added to the standard wells and serum sample wells, respectively. The plate was covered with sealing tape and incubated at 37°C for 30 min. The washing solution was added 5 times manually and incubated for 30 s. Fifty microliters of conjugate reagent (horseradish-peroxidase) was added to the plate except for blank wells, and incubated at 37°C for 30 min. After washing 5 times, 50 μL of chromogenic substrate solutions A and B were added, and the plate was incubated at 37°C in the dark for 10 min. The reaction was stopped by the addition of stop solution, and the plate was read within 15 min on a microplate reader (BioTek Instruments, USA) at a wavelength of 450 nm.

### RNA extraction and quantitative real-time PCR

Total RNA from the brain (whole brain excluding hypothalamus), hypothalamus, liver, ovary, and thymus of hamsters was extracted using an RNAiso Plus kit (TaKaRa, Otsu, Japan). The relative quantity and integrity of total RNA were determined by 1% agarose gel electrophoresis (*U* = 120 V; 10 min). The absorbance of total RNA at 260/280 nm was measured using a Nanodrop 2000 spectrophotometer (Nanodrop Technologies, Wilmington, DE, USA) to measure the purity and concentration of RNA. Total RNA was reversed transcribed into single-strand cDNA using the Prime Script RT kit (TaKaRa, Otsu, Japan).

Primer pairs were purchased from Sangon (Shanghai, China) and are shown in **Supplementary Table S1**. Since *β-actin* was stably expressed in multiple organs (the hypothalamus, preoptic area, hippocampus, liver, ovary, and thymus) and was suitable to study the oscillation pattern of circadian rhythm of target genes, so we used *β-actin* as the reference gene (53–57). TB^®^ *Premix Ex Taq*™ (Tli RNaseH Plus) (TaKaRa, Otsu, Japan) was used for qRT-PCR. The total volume of the mixed reactants was 10 μL, which included 0.2 μL each of forward and reverse primers, 1 μL cDNA templates, 5 μL TB Premix Ex Taq, and 3.6 μL ddH_2_O. The following thermal reaction conditions were used for qRT-PCR: pre-denaturation at 95°C for 3 min, followed by 40 cycles of 95°C for 10 s and 56-65°C for 15 s. A melting curve was generated from 65°C to 95°C. Each sample was run in triplicate, and the results were averaged. The mRNA levels of the target genes were calculated using the 2^−ΔΔCT^ method (58).

### Statistical analyses

All data analyses were performed in *Cosinor. Online* (https://cosinor.online/app/cosinor.php), R (version 4.1.1), and SPSS 20.0 software. All data were used to verify the homogeneity of variance and normality using Levene tests and Shapiro-Wilk tests, respectively. One-way ANOVA was used to examine the variations in behavior, physiology, and expression levels of genes between the six time points. Box-Cox transformation was used to improve the normality and homogeneity of variance of non-normally distributed data (58). Daily rhythms of behavior, physiology, and gene expression were assessed using the cosinor method (59). Behavior, physiology, and gene expression were considered to exhibit rhythmicity when they had *P*-value < 0.3 by cosinor analysis and *P* < 0.05 by ANOVA (6, 60, 61). The correlation of gene expression between behavior and physiology was assessed using the Spearman correlation test (*r*). Figures were drawn in MATLAB (Math Works, Natick, MA, USA), GRAPH PRISM 7.0, Origin Pro 2017 (3D spatiotemporal path reconstructions), and RStudio. Data are presented as means ± SEM.

## Results

### Rhythmicity of circadian behaviors

#### Sleep-wake cycle

**Figure 1:**
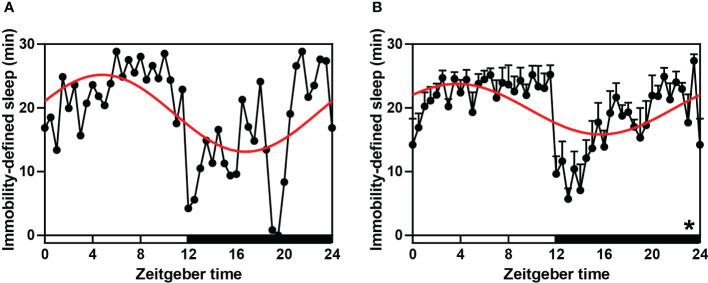
Sleep-wake rhythm (immobility-defined sleep) in hamsters during the daily light-dark cycle. **(A, B)** A single (randomly selected) and average immobility-defined sleep detected using IR Actimeter in hamsters. The red curve represents the cosinor fit curve. White and black represent the light and dark, respectively. In sleep, per 30-min interval is shown. An asterisk (*) indicates that the behavior has a daily rhythm (both *P* < 0.05 and *P*-value < 0.3). Data are means ± SEM.

To test the sleep-wake rhythm of striped hamsters under a daily light-dark cycle, we used IR to detect their sleep characteristics. According to ANOVA and cosinor analyses, the sleep-wake cycle of any single hamster (*P*-value < 0.001, **Figure 1A**, **Supplementary Table S2**) or the average of all hamsters (both *P* < 0.001 and *P*-value < 0.001, **Figure 1B**, **Supplementary Table S2**) exhibited significant 24 h periodic oscillations (**Supplementary Table S2**). ANOVA analysis indicated that the average sleep duration of hamsters in the night phase was significantly lower than during the daytime, and the activity intensity was significantly increased.

#### Open field and elevated plus maze

**Figure 2:**
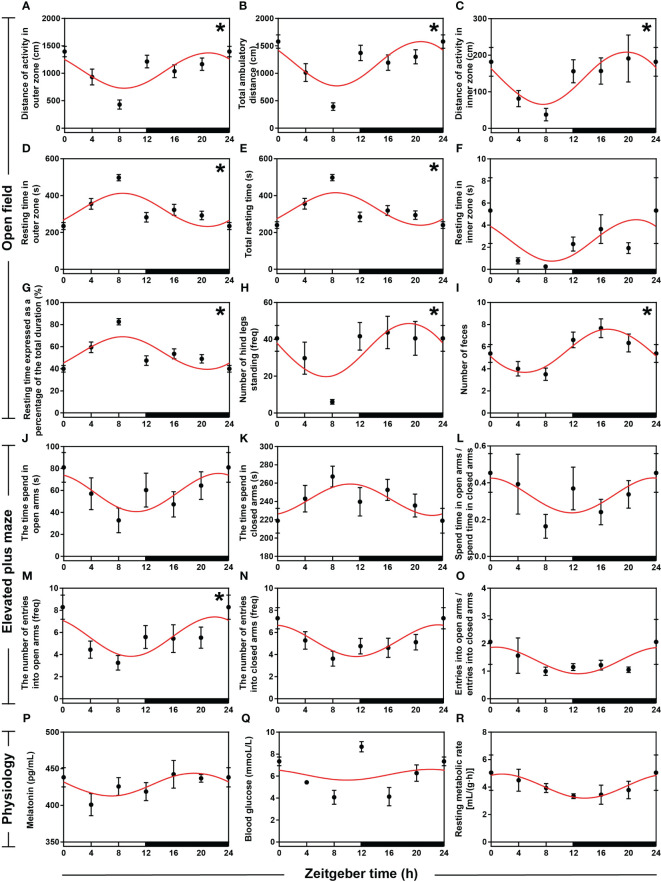
Rhythmicity of circadian behaviors and physiologies in hamsters during the daily light-dark cycle. **(A–I)** The activity rhythms of hamsters in the open field (OF) with an interval of 4 h during the day. **(J–O)** The activity rhythms of hamsters in the elevated plus maze (EPM) with an interval of 4 h during the day. **(P)** Melatonin (MT) levels in serum with an interval of 4 h during the day. **(Q)** Sugar levels in blood with an interval of 4 h during the day. **(R)** Resting metabolic rate (RMR) with an interval of 4 h during the day. The red curve represents the cosinor fit curve. White and black represent the light and dark, respectively. An asterisk (*) indicates that the behavior has a daily rhythm (both *P* < 0.05 and *P*-value < 0.3). Data are means ± SEM.

To examine the behavioral rhythm of hamsters within 24 h, we further performed open field (OF) and elevated plus maze (EPM) tests. We randomly selected a hamster to plot the movement paths at ZT0, ZT4, ZT8, ZT12, ZT16, and ZT20 in the OF (**Supplementary Figure S2**), and the results showed that activity was lowest at ZT8 and highest at ZT0. Analyses of OF indicators using ANOVA and cosinor showed significant daily rhythm in eight indicators (all *P* < 0.05 and *P*-value < 0.3, **Figures 2A–E, G–I**, **Supplementary Table S2**) except the resting time of hamsters in the inner zone (*P* > 0.05, **Figure 2F**, **Supplementary Table S2**). The distance of activity in the outer zone (DAOZ), the distance of activity in the inner zones (DAIZ), total ambulatory distance (TAD), the resting time in the inner zone (RTIZ), the number of hind legs standing (NHLS), and the number of feces (NF) reached lowest points during the daytime and the highest points at night. However, the rest time in the outer zone (RTOZ), total rest time (TRT), and the rest time as a percentage of the total duration [RT (%)] reached their lowest points at night and their highest points during the daytime. Among the six indicators measured in the EPM test (**Figures 2J–O**, **Supplementary Table S2**), there was significant rhythmicity only in the number of entries into the open arms (both *P* < 0.05 and *P*-value < 0.3, **Figure 2M**, **Supplementary Table S2**), and the peak occurred at night.

### Rhythmicity of circadian physiologies

To investigate the characteristics of circadian rhythms in hamsters, we measured serum melatonin (MT) levels, blood sugar levels, and resting metabolic rate (RMR) during the day. ANOVA and cosinor analyses indicated that MT levels in serum (**Figure 2P**, **Supplementary Table S2**), blood sugar levels (**Figure 2Q**, **Supplementary Table S2**), and RMR (**Figure 2R**, **Supplementary Table S2**) did not have strict daily rhythms. However, cosinor analysis showed significant cosinor rhythmicity in serum MT levels and RMR, with both peaks occurring at night. ANOVA analysis revealed significant time-point differences in blood sugar levels, with peaks occurring at the alternation between day and night.

### Expression patterns of circadian genes

**Figure 3:**
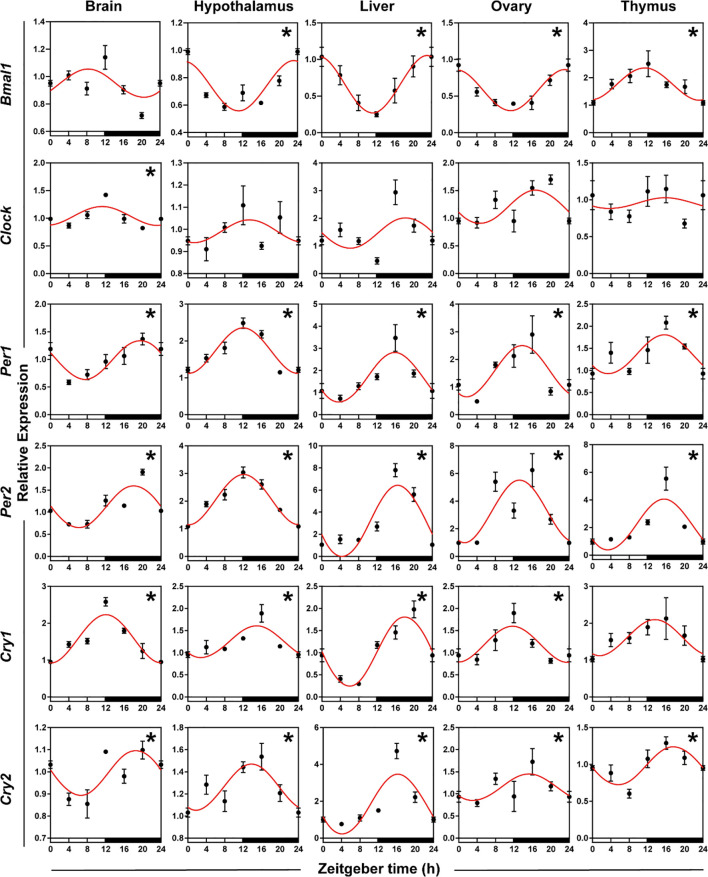
Rhythmicity of circadian genes in hamsters during the daily light-dark cycle. The red curve represents the cosinor fit curve. White and black represent the light and dark, respectively. An asterisk (*) indicates that the gene has a daily rhythm (both *P* < 0.05 and *P*-value < 0.3). Data are means ± SEM.

To identify the daily rhythmic expression of clock genes in the central and peripheral tissues of striped hamsters, we used qRT-PCR to detect the relative expression levels of six clock genes (*Bmal1*, *Clock*, *Per1*, *Per2*, *Cry1*, and *Cry2*) in five tissues (brain, hypothalamus, liver, ovary, and thymus). Statistical analyses, including cosinor and ANOVA, suggested that the expression of the six clock genes in the five tissues exhibited daily rhythms, except *Bmal1* in the brain, *Cry1* in the thymus, and *Clock* in the hypothalamus, liver, ovary and thymus (**Figure 3**, **Supplementary Table S3**, both *P* < 0.05 and *P*-value < 0.3). The expression patterns of *Bmal1* in the hypothalamus, liver, and ovary were similar but contrasted the patterns in the thymus, with peaks occurring at the interphase of day and night, respectively. *Clock* gene expression patterns were roughly similar in the hypothalamus and ovary and roughly similar in the brain, liver, and thymus. The peaks of *Clock*, *Per1*, *Per2*, *Cry1*, and *Cry2* mRNA in the five tissues appeared at the interphase of day and night. Organ differences were found for six clock genes. Phase differences were most familiar, which meant that the nadir and peak of gene expression in one organ appeared a few hours later or earlier than the other peaks, such as *Bmal1* in the hypothalamus and thymus, *Clock*, *Per1*, and *Per2* in the hypothalamus and liver, and *Cry1* in the brain and liver.

### Expression patterns of genes influenced by circadian genes

#### Expression patterns of melatonin receptor genes

**Figure 4:**
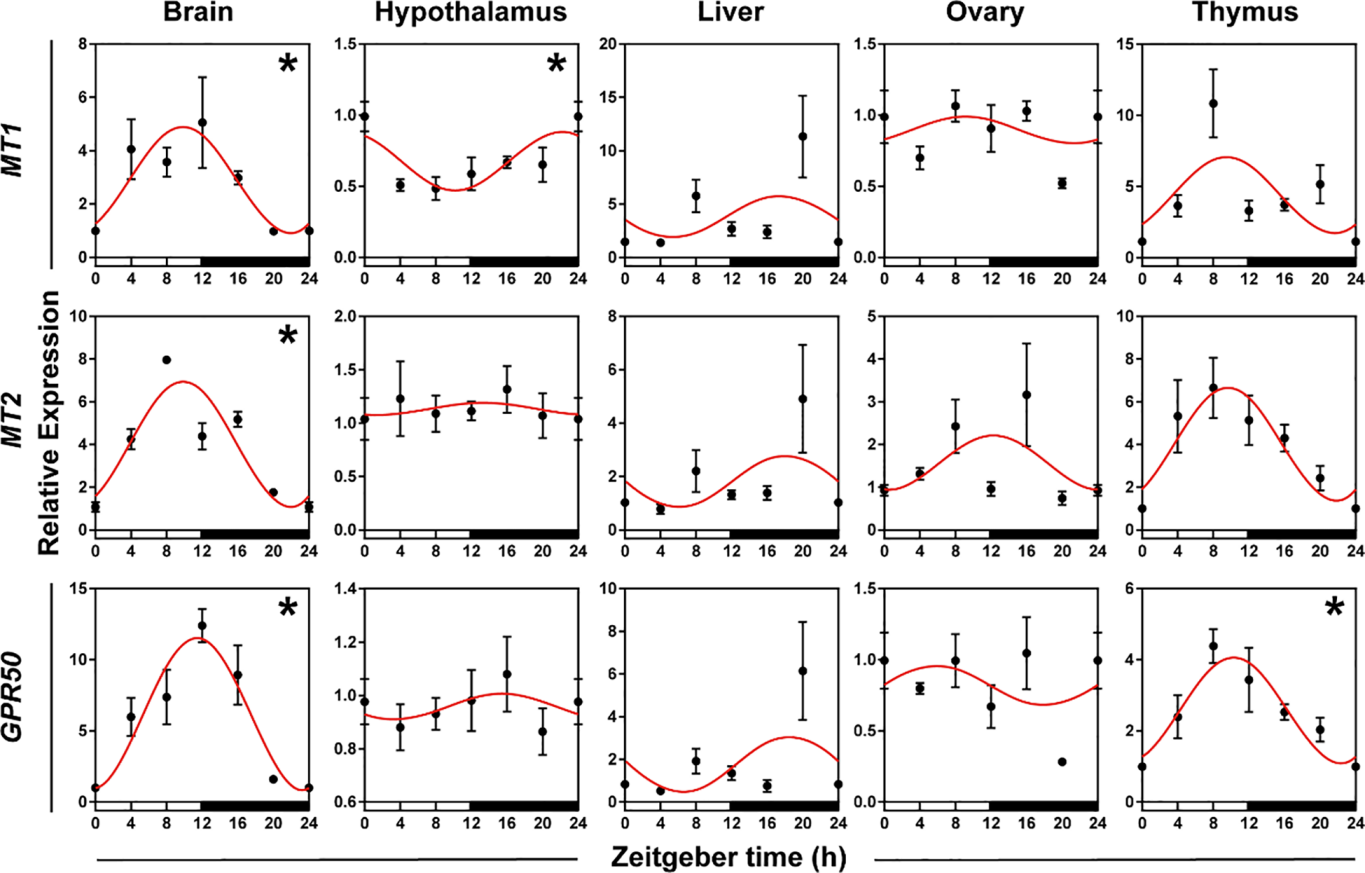
Rhythmicity of melatonin receptor genes in hamsters during the daily light-dark cycle. The red curve represents the cosinor fit curve. White and black represent the light and dark, respectively. An asterisk (*) indicates that the gene has a daily rhythm (both *P* < 0.05 and *P*-value < 0.3). Data are means ± SEM.

To validate whether melatonin receptor genes had daily rhythms in five organs, we used qRT-PCR to detect the expression patterns of three melatonin receptor genes (*MT1*, *MT2*, and *GPR50*). ANOVA and cosinor analyses showed that *MT1*, *MT2*, and *GPR50* in the brain, *MT1* in the hypothalamus, and *GPR50* in the thymus displayed daily rhythmic expression (**Figure 4**, **Supplementary Table S3**, both *P* < 0.05 and *P*-value < 0.3). The rhythmic melatonin receptor genes in the brain and thymus showed an acrophase at the interphase of day and night, except hypothalamic *MT1* exhibited an acrophase during the dark period. The expression patterns of melatonin receptor genes were similar in the brain and thymus, and the genes in the brain and thymus peaked in phase approximately 8 h earlier the genes in the liver.

#### Gene expression patterns involved in general metabolism

**Figure 5:**
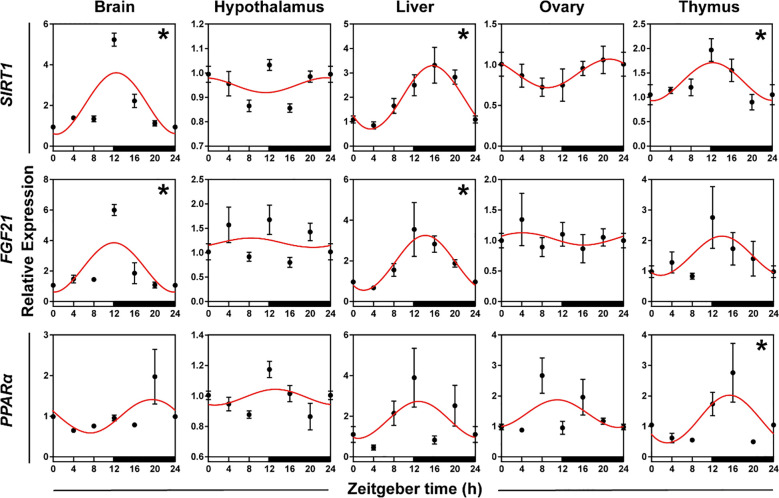
Rhythmicity of genes involved in the general metabolism of hamsters during the daily light-dark cycle. The red curve represents the cosinor fit curve. White and black represent light and dark, respectively. An asterisk (*) indicates that the gene has a daily rhythm (both *P* < 0.05 and *P*-value < 0.3). Data are means ± SEM.

To verify whether genes involved in metabolism displayed daily rhythms, we used qRT-PCR to test the expression patterns of *SIRT1*, *FGF21*, and *PPARα* in five organs. The daily expression profiles of the genes involved in metabolism showed that *SIRT1* in the brain, liver, and thymus, *FGF21* in the brain and liver, and *PPARα* in the thymus exhibited significant daily rhythms, with peak expression occurring at the interphase of day and night or at night. The peak phase of genes in the liver and thymus were delayed 1-4 h compared to the peaks in the brain (**Figure 5**, **Supplementary Table S3**, both *P* < 0.05 and *P*-value < 0.3).

### Correlation of gene expression between behavior and physiology

**Figure 6:**
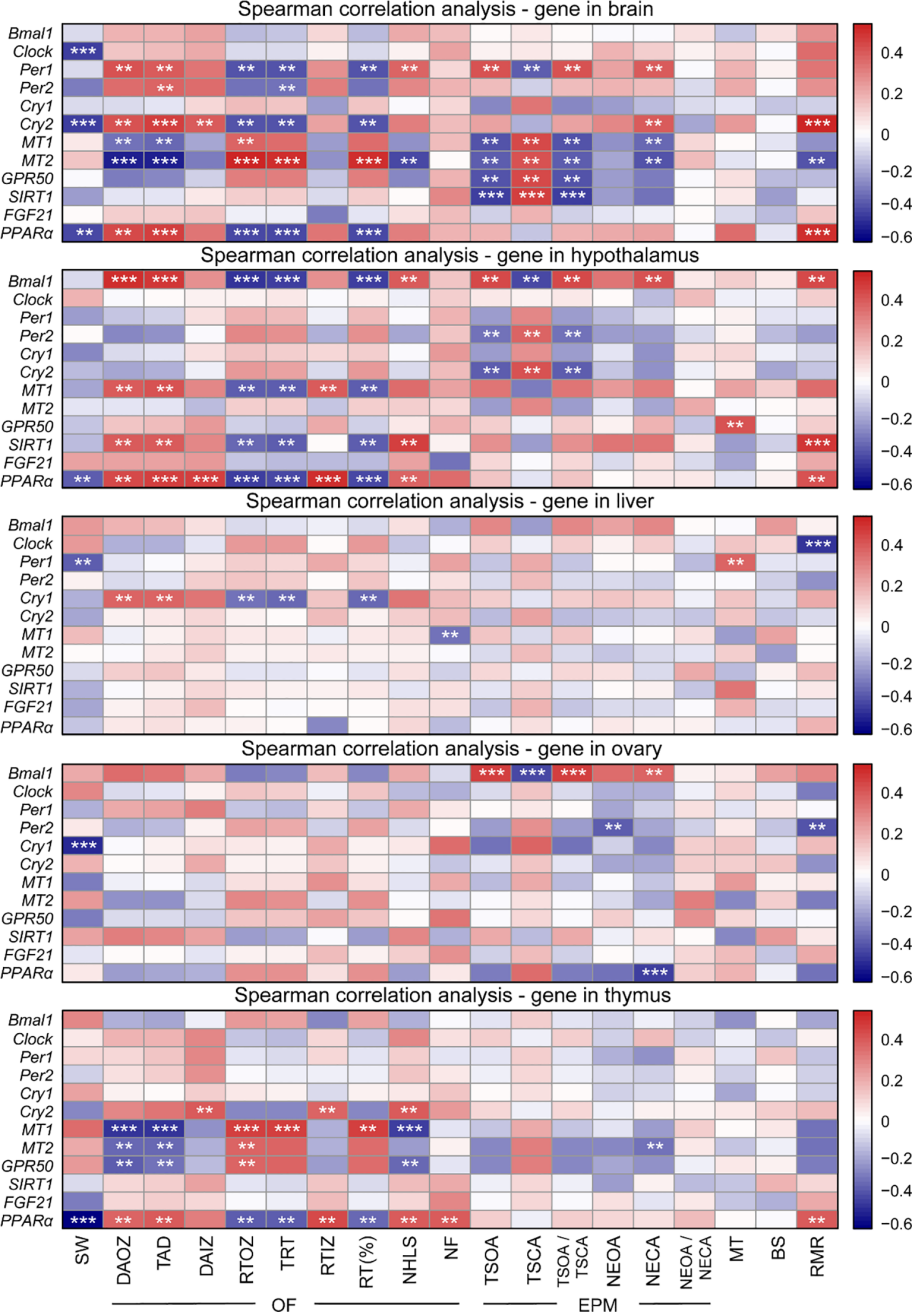
Heatmap showing Spearman correlation of gene expression between behavior and physiology. OF, open field; EPM, elevated plus maze; SW, sleep-wake; DAOZ, distance of activity in the outer zone; TAD, total ambulatory distance; DAIZ, distance of activity in the inner zones; RTOZ, resting time in the outer zone; TRT, total resting time; RTIZ, resting time in the inner zone; RT (%), resting time as a percentage of the total duration; NHLS, number of hind legs standing; NF, number of feces; TSOA, the time spend in open arms; TSCA, the time spend in closed arms; TSOA/TSCA, the ratio of spend time in open arms to spend time in closed arms; NEOA, the number of entries into open arms; NECA, the number of entries into closed arms; NEOA/NECA, the ratio of entries into open arms to entries into closed arms; MT, melatonin; BS, blood sugar; RMR, resting metabolic rate. **P < 0.01, ***P < 0.001.

To confirm whether clock genes, melatonin receptor genes, and genes involved in metabolism in central and peripheral tissues regulated the daily rhythm of behavior and physiology, we investigated the association of genes with behavior and physiology using Spearman correlation analysis (**Figure 6**). In the brain, *Clock*, *Cry2*, and *PPARα* mRNA were negatively correlated with the sleep-wake cycle; *Per1*, *Per2*, *Cry2*, *MT1*, *MT2*, and *PPARα* mRNA were correlated with OF; *Per1*, *Cry2*, *MT1*, *MT2*, *GPR50*, and *SIRT1* were correlated with EPM; *Cry2* and *PPARα* mRNA were positively correlated with RMR, and *MT2* mRNA was negatively correlated with RMR. In the hypothalamus, *PPARα* was negatively correlated with the sleep-wake cycle; the expression of four genes (*Bmal1*, *MT1*, *SIRT1*, and *PPARα*) were correlated with OF; three genes (*Bmal1*, *Per2*, and *Cry2*) mRNA were correlated with EPM; *GPR50* mRNA was positively correlated with serum MT levels; three genes expression levels (*Bmal1*, *SIRT1*, and *PPARα*) were positively correlated with RMR. In the liver, the expression of *Per1* was negatively correlated with the sleep-wake cycle, but positively correlated with serum MT levels; *Cry1* and *MT1* mRNA were correlated with OF; *Clock* mRNA was negatively correlated with RMR. In the ovary, *Cry1* mRNA was negatively correlated with the sleep-wake cycle; the expression of *Bmal1*, *Per2*, and *PPARα* were correlated with EPM; *Per2* mRNA was negatively with RMR. In the thymus, *PPARα* mRNA was negatively correlated with the sleep-wake cycle; *Cry2*, *MT1*, *MT2*, *GPR50*, and *PPARα* mRNA were corrected with OF; *MT2* mRNA was negatively corrected with EPM; *PPARα* mRNA was positively correlated with RMR (all *P* < 0.01). However, there was no correlation between blood sugar and genes in the five organs (all *P* > 0.05).

## Discussion

This study is the first investigation of the rhythmicity of behavioral, physiological, central and peripheral tissue genes (clock genes, melatonin receptor genes, and genes involved in metabolism) in striped hamsters. The present study found that hamsters had significant circadian rhythms, most genes were expressed rhythmically in tissues, and these genes significantly correlated with behavior and physiology. These results suggest that central and peripheral clock may regulate the daily rhythmicity of behavior, physiology, and metabolism in striped hamsters.

### Circadian behaviors

Several methods are used to monitor sleep-wake in small rodents, primarily traumatic electroencephalography (EEG) and electromyography (EMG) (62, 63). However, the surgical procedure of electrode implantation is harmful to animal health, time-consuming and laborious, and not conducive to the large-scale screening of animal sleep patterns (64–66). The present study successfully traced the sleep-wake cycle of unconstrained small rodents using an infrared open field combined with computer analysis. We examined the sleep-wake behavior of striped hamsters and found that their daytime activity was significantly less than their nighttime activity. Peak activity occurred at the alternation of day and night and showed an activity cycle of nearly 24 h, which is consistent with previous results in mice tested using EEG (52). This result demonstrated that our new method was scientific and reliable and confirmed that the striped hamster has the characteristics of nocturnal animals.We used the open field and elevated plus maze to measure the circadian activity rhythms of hamsters from their level of movement and preference for closed environments, respectively. Our results demonstrated that striped hamsters had an increased defecation number and decreased resting time at night, and the most active time occurred at ZT20 (4:00). The striped hamsters in the wild are most active at 20:00-22:00 and 4:00-6:00 in different seasons (67, 68). Our laboratory research showed that the most frequent activity occurred 4 h before the light turned on, which was roughly the same as the peak activity period in the wild environment. This result indicated that indoor domestication may not change its rhythmic behavior, and clock genes primarily regulated hamster behavior.

Photoperiod, temperature, rainfall, and grazing are distributed in the wild environment, which is quite different from the indoor environment. Although the study shows that the balance between entrainment and masking processes appears to generate a temporal niche gradient (69), we argue that it remains significant to distinguish whether hamsters are diurnal or nocturnal. The striped hamster is the dominant species living on farmlands and grasslands. It has a strong reproductive ability and is extremely destructive to these areas. The activity pattern of the circadian rhythm of striped hamsters was detected to identify the peak of their activity and capture them to achieve rodent extermination and provide a theoretical basis for controlling rodent damage.

### Circadian physiologies

Metabolic activity is maintained by the energy produced and stored in metabolism, such as the resting metabolic rate (RMR) (70). The present study found that RMR decreased during sleep and increased during activity in hamsters in indoor environments, primarily due to the need to expend more energy to maintain body temperature at night. Bao et al. (2022) measured the basal metabolic rate (BMR) of striped hamsters in the wild and found that these animals did not adapt well to the arid desert environment (71). The striped hamster in the wild is characterized by high body temperature and high BMR (2.20 ± 0.09 mL O_2_ g^−1^ h^−1^ in the thermoneutral region), which is related to its distribution in high latitudes. High oxygen consumption makes it better adapted to the cold field environment and matches its nocturnal characteristics (72). Melatonin is a neuroendocrine hormone that affects the growth, and reproduction of mammals (73, 74). During the night (especially between 11:00 p.m. and 5:00 a.m.), blood melatonin levels increase 3-10 times and the secretion of melatonin peaks (75). Mice lacking melatonin receptors (such as MT1) had slightly higher daytime activity than mice without melatonin receptors deficiency (76, 77), which suggests that melatonin may influence activity rhythms by acting on receptors. Melatonin plays an important role in regulating the expression of clock genes, which synchronize central and peripheral oscillators (78, 79). The peak secretion of melatonin was primarily concentrated at night, which indicated that melatonin played a pivotal role in initiating circadian rhythm and responding to changes in photoperiod. These results are consistent with a previous study (80). Blood sugar levels showed no daily rhythm, which indicated that it was primarily influenced by the timing of food intake (81).

### Expression patterns of rhythmic genes

#### Clock genes

Clock genes in the SCN interact with each other to regulate circadian rhythm (8). Most of the clock genes in the hypothalamus had rhythmic characteristics in the present study, which demonstrated that the central regulatory system played a crucial role in regulating daily rhythm and indicated the stability of endogenous clock gene regulation in the SCN (82). By comparing the expression patterns of clock genes in the hypothalamus and peripheral tissues, we found that the phase delay with the peripheral clock was likely due to the pacemaker in the SCN synchronizing clock genes in the peripheral tissues with the master oscillator and external time (83). We also found that circadian rhythms in rodents were tissue-specific, which is consistent with previous studies (7, 15). Notably, *Clock* mRNA showed weaker rhythmicity in the hypothalamus, liver, ovary, and thymus, but this phenomenon was not the first pattern to be discovered. For example, the expression of *Clock* was rhythmless in Syrian hamsters under long photoperiod (84) and did not oscillate in *Spalax* (85). However, these genes without obvious rhythm would not necessarily affect endogenous activity regulation because of the complementary function of positive arms (*Bmal1* and *Clock*) and negative arms (*Pers* and *Crys*) (59).

#### Melatonin receptor genes

Melatonin is regulated by the endogenous clock, and it is an important neuroendocrine output of the clock (74). However, melatonin feeds back on the SCN by resetting the circadian clock to regulate its function (86, 87). Our data showed that circadian expression patterns of melatonin receptor genes were similar in the same tissue, which further indicated the tissue specificity of rodents. However, *MT1* mRNA oscillations were observed only in the hypothalamus, which suggested that the role of melatonin in the SCN was primarily attributable to the MT1 receptor with a smaller role for MT2, which is consistent with previous findings that the MT2 receptor was not necessary for the melatonin response to circadian changes (88). The expression of *MT1*, *MT2*, and *GPR50* in the brain increased at dusk, which suggests that melatonin receptors synthesis undergoes periodic changes in the circadian cycle (89). The expression of *GPR50* was markedly rhythmical in multiple organs (the brain and thymus), which suggested that *GPR50* played a conservative role in neuroendocrine regulation and a potential role in coordinating physiological responses of the central nervous system and surrounding tissues.

#### Genes involved in general metabolism

The expression patterns of most genes involved in metabolism in mammals are subject to diurnal variations controlled by the circadian clock (90). Genes involved in metabolism were more rhythmic in the brain, liver and thymus in the present study. The rhythmic expression and activation of metabolic pathways are primarily related to the coordination of clock genes (*Bmal1*, *Pers*, and *Crys*) in the liver and adipose tissue (91). The peak expression of *SIRT1* and *FGF21* in the liver appeared at night with a significant rhythm, which suggested that clock genes might regulate energy metabolism by regulating *SIRT1* and *FGF21* mRNA in the liver (30). Tissue-specific clocks work differently in the liver than other organs. For example, loss of *Bmal1* in the liver of mouse led to an imbalance in the rhythm of key metabolic genes (92). Therefore, the circadian clock favors glucose stability during feeding and fasting by influencing metabolic processes (36). Notably, SIRT1 generates a negative feedback loop by modulating CLOCK/BMAL1 activity, which modulates the circadian clock by interacting with BMAL1, CLOCK, and PER2 (32, 33, 93). The rhythmic expression of *PPARα* in the liver is transcriptionally regulated by clock genes, which activates *Bmal1* expression in the liver (94). Notably, the poor rhythm of *PPARα* in the liver in the present study might be due to species differences.

### Correlation of genes with behavior and physiology

The SCN is the primary circadian pacemaker, and it sends projections to important sleep regulatory nuclei (e.g., ventrolateral preoptic nucleus). Rats and mice showed disturbed sleep time after SCN injury (95), and sleep state affected the activity of SCN neurons. Prolonged wakefulness affected the expression of clock genes in the cerebral cortex and upregulated *Per1* and *Per2* mRNA among mouse strains (96). *Clock*, *Per1*, *Cry1* and *Cry2* were significantly negatively correlated with sleep time in central and peripheral tissues, which suggested that clock genes played a crucial role in regulating sleep-wake and sleep homeostasis control (2). There was a significant negative correlation between *PPARα* mRNA in the brain, hypothalamus, and thymus and sleep duration, which may be because *PPARα* activation promotes increased wakefulness while reducing sleep (97). In fact, *PPARα* can directly regulate the expression of *Bmal1* in the liver and interact with the circadian clock (98). For example, an altered *Bmal1* rhythmic oscillation pattern was found in the liver of *PPARα*-null mice, while the *PPARα* circadian expression was abolished in the liver of *Bmal1* knockout mice (98). The association of both circadian clock and *PPARα* with sleep-wake in the present study may further support the interaction between them and their plasticity in regulating sleep. In addition, sleep-wake rhythms depend on gonadal function, and gonadal hormones influence sleep-wake in gonadectomized mice (99). Ovarian *Cry1* was significantly negatively correlated with sleep-wake, suggesting that the ovary may be an important organ for sleep regulation. There was no correlation between thymic clock genes and sleep-wake, but it did not mean that the thymus was not involved in the regulation of sleep.

Deficiency of the circadian proteins *Clock*, *Cry1*, and *Cry2* alters exploratory behavior in mice (100). In this study, we found that clock genes in the central and peripheral tissues significantly positively correlated with the hamster’s movement distance in the open field, and positively correlated with the number of entries into the open arms in the elevated plus maze. These results suggest that it may be under the regulation of clock genes, and the exploration of the animal behavior and habit of novelty showed normal movement. The melatonin receptors *MT1*, *MT2*, and *GPR50* in the brain, hypothalamus, and thymus were related with exploration behavior in hamsters. In fact, MT1 receptor knockout mice showed anxiety behavior with circadian variation, and MT1 regulates circadian rhythm by participating in melatonin to acutely inhibit SCN discharge rate (101). Most importantly, serotonin (5-hydroxytryptamine; 5-HT) neurotransmission is altered upon inactivation of MT1 (102). 5-HT controls sleep-wake and anxiety behaviors (103), and the absence of MT1 makes it associated with circadian imbalance and multiple behaviors (102). Therefore, we hypothesized that melatonin may alter 5-HT neurotransmission by acting on receptors, which in turn regulate the behavior of hamsters.

*SIRT1* is a key regulator of metabolic processes and longevity (104). It causes the expression of several core clock genes, such as *Cry1*, *Bmal1*, and *Per2* (105), and serves as an important factor in the regulation of hepatic circadian rhythms (33, 106). Its activity is upregulated by melatonin through the membrane receptor pathway (107). *SIRT1* knockout mice showed increased anxiety behavior and decreased activity (108). This study found that the daily rhythm of *SIRT1* was significantly related to open field behavior in multiple tissues, suggesting that melatonin may regulate circadian clock by affecting SIRT1 mRNA, thereby affecting the daily rhythm patterns of anxiety behavior and exploratory behavior. In addition, the expression of *Bmal1* and *SIRT1* in the hypothalamus were significantly positively correlated with RMR. The central circadian clock regulates *SIRT1* activity via the rhythmic biosynthesis of NAD+ to regulate the metabolic level of the organism. Therefore, we hypothesized that *Bmal1* may affect resting metabolic rate of hamsters by regulating *SIRT1*. In summary, *SIRT1* may interact with the clock genes and melatonin, and genes involved in metabolism may play a key role in the stabilization of circadian rhythm in hamsters.

### Comparison of *Cricetulus barabensis* with other rodents

In this study, *Cricetulus barabensis* had circadian behavior rhythm in the 12L:12D light-dark cycle, and there were stable circadian changes in the central and peripheral core clocks. This phenomenon had also been found in other rodents. For example, under the 12L:12D photoperiod, the expression levels of multiple clock genes in six peripheral tissues (liver, kidney, spleen, testis, thymus, and blood) were different in mice, and the circadian rhythm was obvious in some tissues (15). The expression patterns of the six circadian clock genes in five tissues (testis, kidney, liver, spleen, and heart) of the three hamsters were rhythmic and varied among species (7). *Microtus arvalis* had hyperactivity and feeding rhythms, and circadian genes were expressed in the central (SCN) and metabolic organs (liver) (109). Brandt’s voles and Mandarin voles showed a behavioral pattern of low daytime activity and high nocturnal activity under constant darkness and 12L:12D day-night cycle, similar to the activity pattern of striped hamsters (59).

### Limitation of this study

There were several limitations in this study. First, this experiment was always maintained in a 12L:12D environment, and did not examine animal behavior and gene expression profiles in continuous darkness, so endogenous profiles of any rhythmic fluctuations could not be assessed. To thoroughly understand the circadian rhythm of striped hamsters, more studies in the field and indoor environment are needed (110). Different dark cycle treatments should be further used to examine the differences between hamsters in the rhythm disorder state and normal rhythm state at the level of individuals and cells, and clarify the regulatory mechanism of circadian rhythm changes. Second, the present findings established correlations between gene expression and behavioral and physiological parameters. However, these analyses remain descriptive, and the causal roles of these factors in behavior are not demonstrated. Future research should be devoted to exploring the role of genes in regulating hamster behavior, movement, and metabolic rate through gene knockout or gene silencing, and looking for causal effects between them. Third, since the sample size of each group in this study was small (n = 6), in order to increase the number of tested hamsters and reduce errors caused by individual differences, open field and elevated plus maze were examined in all hamsters. However, this means that re-exposure to the testing arena resulted in reduced activity in the animals (111). To minimize behavioral changes induced by repeated exposure, the timing of hamsters’ first exposure to the open field and elevated plus maze was randomized. Future studies should increase the sample size per group (e.g., more than 10 animals) and examine each animal’s behavior only at a single time point to eliminate adaptation in the testing field due to re-exposure.

## Conclusions

The results of this study indicate that the expression patterns of clock genes, melatonin receptor genes, and genes involved in general metabolism are highly rhythmic in central and peripheral tissues. Clock genes may directly regulate melatonin receptor genes and genes involved in general metabolism. The central clock system may link to the peripheral clock system to jointly maintain circadian behavior and energy metabolism in small rodents. Our results provide important evidence that the circadian clock, melatonin, and metabolism may regulate host behavior and clues for pest control.

## Data availability statement

The original contributions presented in the study are included in the article/**Supplementary Material**. Further inquiries can be directed to the corresponding author. Some or all datasets generated during and/or analyzed during the current study are not publicly available but are available from the corresponding author on reasonable request.

## Ethics statement

The animal study was approved by the Ethics Committee of Qufu Normal University. The study was conducted in accordance with the local legislation and institutional requirements.

## Author contributions

HZhu: Data curation, Formal Analysis, Investigation, Methodology, Software, Visualization, Writing – original draft. MW: Formal Analysis, Funding acquisition, Methodology, Supervision, Writing – review & editing. JM: Investigation, Methodology, Writing – review & editing. XY: Investigation, Methodology, Writing – review & editing. QX: Investigation, Methodology, Writing – review & editing. YZ: Investigation, Writing – review & editing. HZha: Investigation, Writing – review & editing. XW: Investigation, Writing – review & editing. HX: Funding acquisition, Methodology, Supervision, Writing – review & editing. JX: Funding acquisition, Methodology, Supervision, Writing – review & editing. LC: Methodology, Supervision, Writing – review & editing. LX: Funding acquisition, Methodology, Supervision, Writing – review & editing.
