# Supplementary material for: Behavior and physiology in female Cricetulus barabensis are associated with the expression of circadian genes
Source: Front Endocrinol (Lausanne). 2024 Jan 4;14:1281617. doi: 10.3389/fendo.2023.1281617 (PMC10875996; doi:10.3389/fendo.2023.1281617)
Supplement: Supplementary file 1 [file Image_1.pdf]

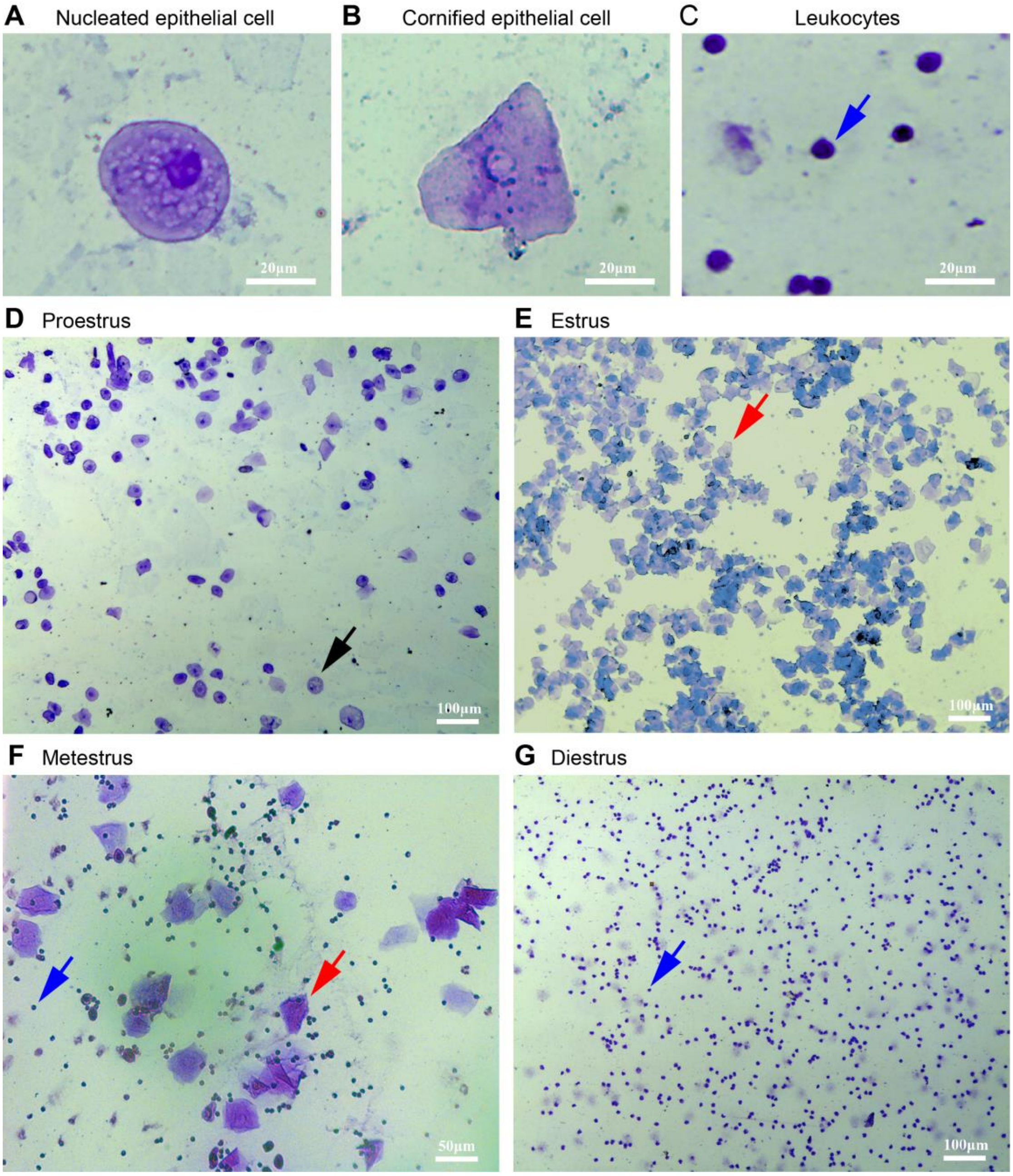

Figure S1. Vaginal smears of female *Cricetulus barabensis* in estrous cycle. **(A)** Nucleated epithelial cells. **(B)** Cornified squamous epithelial cells. **(C)** Leukocytes. **(D)** Proestrus. **(E)** Estrus. **(F)** Metestrus. **(G)** Diestrus. Black arrow in **(D)** indicate nucleated epithelial cells. Red arrows in **(E)** and **(F)** highlight representative cornified squamous epithelial cells. Blue arrows in **(F)** and **(G)** point to leukocytes.
