# Supplementary material for: Behavior and physiology in female Cricetulus barabensis are associated with the expression of circadian genes
Source: Front Endocrinol (Lausanne). 2024 Jan 4;14:1281617. doi: 10.3389/fendo.2023.1281617 (PMC10875996; doi:10.3389/fendo.2023.1281617)
Supplement: Supplementary file 2 [file Image_2.pdf]

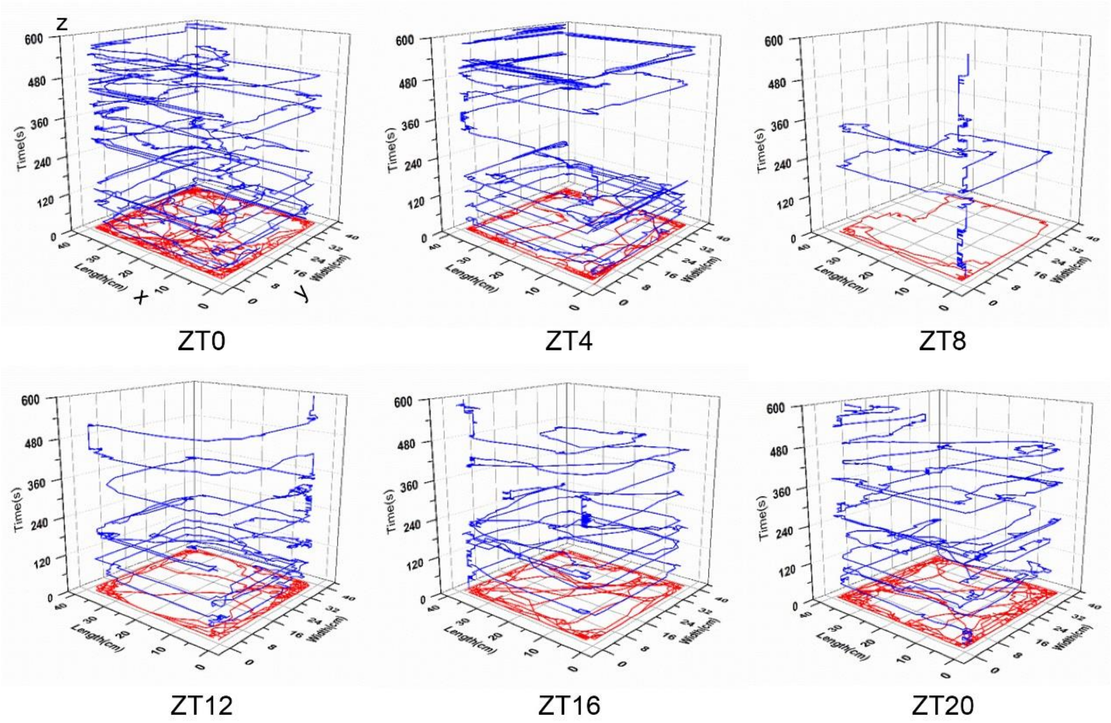

Figure S2. Spatiotemporal behavioral phenotyping of a single hamster in open field at ZT0, ZT4, ZT8, ZT12, ZT16, and ZT20 for 10 minutes. Red curves indicate the plane plan of the hamster's movement path, and blue curves represent the stereogram of the hamster's movement track. x axis represents the length of the open field (40 cm), y axis represents the width of the open field (40 cm), and z axis represents the time axis (600 s).
