# Supplementary material for: Behavior and physiology in female Cricetulus barabensis are associated with the expression of circadian genes
Source: Front Endocrinol (Lausanne). 2024 Jan 4;14:1281617. doi: 10.3389/fendo.2023.1281617 (PMC10875996; doi:10.3389/fendo.2023.1281617)
Supplement: Supplementary file 3 [file Table_1.docx]

**Table S1** Primer sequences used in qRT-PCR.

| Target gene | Primer sequence^a^ (5'-3') | Length | Annealing temp. |
| --- | --- | --- | --- |
| *Bmal1* | F: CGGTTCCTTTGTCACACTACG | 209 bp | 60℃ |
|  | R: TGGGATGAGTCCTTGGG |  |  |
| *Clock* | F: TAGGGCTGAAAGACGACG | 258 bp | 58℃ |
|  | R: AAGTGCTGTCTGGGAGGAG |  |  |
| *Per1* | F: GCCTATCGTCCAGTGGGTCT | 95 bp | 63℃ |
|  | R: CTGCCAAGGTCTCTGAAGCG |  |  |
| *Per2* | F: CTTACACCATGGAGCAGGTTGAGG | 166 bp | 60℃ |
|  | R: AACTTGGCGTCACTGAAGGCATC |  |  |
| *Cry1* | F: GGGCTAGGTCTTCTTGCATC | 155 bp | 64℃ |
|  | R: TGCTGACTGTCTCCATGAGC |  |  |
| *Cry2* | F: ACTACATTCGGCGATACCTG | 230 bp | 56℃ |
|  | R: GACAGATGCCAGAAGACAGAGT |  |  |
| *MT1* | F: ACCCAGACTGAAGCCACAGGAC | 112 bp | 61℃ |
|  | R: TGAAGCCACAATGAGCCCGATG |  |  |
| *MT2* | F: CCTCGTCTGGCTCCTCACTCTG | 118 bp | 65℃ |
|  | R: CGTGTACTGTGTGCTGGCTGTC |  |  |
| *GPR50* | F: TGCGAACAACTCTGCCTTCACG | 110 bp | 61℃ |
|  | R: GGGCTGCCAGCACTTTGATCC |  |  |
| *SIRT1* | F: AGTGCTGGCCCAATAGACTTGC | 114 bp | 62℃ |
|  | R: CTGAGTATACCTCCGCGCCATG |  |  |
| *FGF21* | F: CACACCGCAGCCCAGAAAGTC | 125 bp | 62℃ |
|  | R: AGGCGATCCATACAGGGTTCCG |  |  |
| *PPARα* | F: TGTATGGCTGAGAAGACGCTTGTG | 90 bp | 62℃ |
|  | R: CTGGCAGCAGTGGAAGATTCGG |  |  |
| *β-actin* | F: GAGACCTTCAACACCCCAGC | 256 bp | 60℃ |
|  | R: ATGTCACGCACGATTTCCC |  |  |

**Note:** ^a^ F = forward; R = reverse.
