# Supplementary material for: Behavior and physiology in female Cricetulus barabensis are associated with the expression of circadian genes
Source: Front Endocrinol (Lausanne). 2024 Jan 4;14:1281617. doi: 10.3389/fendo.2023.1281617 (PMC10875996; doi:10.3389/fendo.2023.1281617)
Supplement: Supplementary file 4 [file Table_2.docx]

**Table S2** Circadian rhythm characteristics and statistical analysis of behavior and physiology in hamsters reared under the daylight-dark cycle condition.

| Behavior & Physiology | | Cosinor analysis | | | |  | ANOVA |
| --- | --- | --- | --- | --- | --- | --- | --- |
|  |  | Mesor | Amplitude | Acrophase (h) | *P*-value |  | *P* |
| Sleep-wake | IDSH | **18.98** | **5.99** | **4.78** | **<0.001** |  | **—** |
|  | IDSAH | **19.65** | **3.93** | **3.71** | **<0.001** |  | **<0.001** |
| Open filed | DAOZ | **1052.06** | **320.79** | **20.73** | **0.23** |  | **<0.001** |
|  | TAD | **1168.94** | **398.52** | **20.53** | **0.23** |  | **<0.001** |
|  | DAIZ | **136.99** | **71.58** | **19.50** | **0.049** |  | **0.03** |
|  | RTOZ | **325.05** | **88.64** | **8.61** | **0.22** |  | **<0.001** |
|  | TRT | **326.39** | **87.76** | **8.51** | **0.21** |  | **<0.001** |
|  | RTIZ | 2.61 | 1.84 | 20.96 | 0.29 |  | 0.11 |
|  | RT (%) | **54.40** | **14.48** | **8.54** | **0.22** |  | **<0.001** |
|  | NHLS | **34.12** | **14.49** | **19.03** | **0.16** |  | **0.01** |
|  | NF | **5.62** | **1.93** | **17.07** | **0.01** |  | **0.001** |
| Elevated plus maze | TSOA | 58.30 | 17.26 | 22.55 | 0.15 |  | 0.24 |
|  | TSCA | 241.71 | 17.26 | 10.55 | 0.15 |  | 0.24 |
|  | TSOA/TSCA | 0.33 | 0.09 | 23.73 | 0.28 |  | 0.46 |
|  | NEOA | 5.22 | 1.4 | 23.36 | 0.09 |  | 0.07 |
|  | NECA | **5.62** | **1.77** | **21.85** | **0.21** |  | **0.03** |
|  | NEOA/NECA | 1.38 | 0.46 | 0.70 | 0.14 |  | 0.51 |
| Physiology | Melatonin | 428.32 | 15.65 | 18.89 | 0.19 |  | 0.28 |
|  | Blood sugar | 6.12 | 0.48 | 21.83 | 0.91 |  | 0.001 |
|  | RMR | 4.04 | 0.97 | 1.58 | 0.009 |  | 0.57 |

**Note:** Mesor, a rhythm-adjusted mean; Amplitude, a measure of half the extent of predictable variation within one cycle; Acrophase (h), a measure of the time of overall high values recurring in each cycle; IDSH, immobility-defined sleep in a hamster; IDSAH, immobility-defined sleep in all hamsters; DAOZ, distance of activity in outer zone; TAD, total ambulatory distance; DAIZ, distance of activity in inner zones; RTOZ, resting time in outer zone; TRT, total resting time; RTIZ, resting time in inner zone; RT (%), resting time as a percentage of the total duration; NHLS, number of hind legs standing; NF, number of feces; TSOA, the time spend in open arms; TSCA, the time spend in closed arms; TSOA/TSCA, spend time in open arms/spend time in closed arms; NEOA, the number of entries into open arms; NECA, the number of entries into closed arms; NEOA/NECA, entries into open arms/entries into closed arms; RMR, resting metabolic rate. There was a statistical significance when both *P* < 0.3 and *P*-value < 0.5 (in bold).
