# Supplementary material for: Behavior and physiology in female Cricetulus barabensis are associated with the expression of circadian genes
Source: Front Endocrinol (Lausanne). 2024 Jan 4;14:1281617. doi: 10.3389/fendo.2023.1281617 (PMC10875996; doi:10.3389/fendo.2023.1281617)
Supplement: Supplementary file 5 [file Table_3.docx]

**Table S3** Circadian rhythm characteristics and statistical analysis of gene expression in the brain, hypothalamus, liver, ovary, and thymus of hamsters reared under the daylight-dark cycle condition.

| Gene | | Cosinor | | | |  | ANOVA |
| --- | --- | --- | --- | --- | --- | --- | --- |
|  |  | Mesor | Amplitude | Acrophase (h) | *P*-value |  | *P* |
| Circadian gene in the brain | *Bmal1* | 0.95 | 0.10 | 8.24 | 0.40 |  | <0.001 |
|  | *Clock* | **1.00** | **0.17** | **11.30** | **0.27** |  | **<0.001** |
|  | *Per1* | **0.99** | **0.35** | **19.42** | **0.03** |  | **<0.001** |
|  | *Per2* | **1.12** | **0.46** | **18.12** | **0.11** |  | **<0.001** |
|  | *Cry1* | **1.58** | **0.65** | **12.17** | **0.03** |  | **<0.001** |
|  | *Cry2* | **0.99** | **0.10** | **18.48** | **0.21** |  | **<0.001** |
| Circadian gene in the hypothalamus | *Bmal1* | **0.74** | **0.17** | **23.15** | **0.10** |  | **<0.001** |
|  | *Clock* | 0.99 | 0.05 | 13.36 | 0.50 |  | 0.08 |
|  | *Per1* | **1.74** | **0.61** | **11.98** | **0.01** |  | **<0.001** |
|  | *Per2* | **2.07** | **0.89** | **12.2** | **0.003** |  | **<0.001** |
|  | *Cry1* | **1.25** | **0.35** | **14.87** | **0.11** |  | **<0.001** |
|  | *Cry2* | **1.26** | **0.2** | **13.2** | **0.10** |  | **0.001** |
| Circadian gene in the liver | *Bmal1* | **0.66** | **0.39** | **23.18** | **<0.001** |  | **0.001** |
|  | *Clock* | 1.47 | 0.55 | 18.11 | 0.54 |  | <0.001 |
|  | *Per1* | **1.69** | **1.12** | **15.9** | **0.03** |  | **<0.001** |
|  | *Per2* | **3.21** | **3.23** | **16.52** | **0.049** |  | **<0.001** |
|  | *Cry1* | **1.03** | **0.79** | **17.99** | **0.008** |  | **<0.001** |
|  | *Cry2* | **1.86** | **1.62** | **16.32** | **0.08** |  | **<0.001** |
| Circadian gene in the ovary | *Bmal1* | **0.58** | **0.28** | **23.38** | **0.01** |  | **<0.001** |
|  | *Clock* | 1.21 | 0.30 | 16.77 | 0.32 |  | <0.001 |
|  | *Per1* | **1.59** | **0.92** | **13.83** | **0.08** |  | **<0.001** |
|  | *Per2* | **3.25** | **2.27** | **13.25** | **0.10** |  | **<0.001** |
|  | *Cry1* | **1.19** | **0.41** | **11.73** | **0.07** |  | **<0.001** |
|  | *Cry2* | **1.15** | **0.29** | **15.26** | **0.29** |  | **0.04** |
| Circadian gene in the thymus | *Bmal1* | **1.79** | **0.59** | **11.22** | **0.02** |  | **0.02** |
|  | *Clock* | 0.96 | 0.072 | 15.84 | 0.84 |  | 0.17 |
|  | *Per1* | **1.37** | **0.44** | **15.63** | **0.16** |  | **0.001** |
|  | *Per2* | **2.22** | **1.86** | **15.55** | **0.08** |  | **<0.001** |
| Circadian gene in the thymus | *Cry1* | 1.62 | 0.47 | 13.58 | 0.04 |  | 1.17 |
|  | *Cry2* | **0.98** | **0.26** | **17.58** | **0.06** |  | **<0.001** |
| Melatonin receptor gene in the brain | *MT1* | **2.90** | **1.99** | **9.86** | **0.03** |  | **0.01** |
|  | *MT2* | **4.02** | **2.89** | **9.88** | **0.048** |  | **<0.001** |
|  | *Gpr50* | **6.23** | **5.31** | **11.41** | **0.008** |  | **<0.001** |
| Melatonin receptor gene in the hypothalamus | *MT1* | **0.67** | **0.20** | **22.17** | **0.18** |  | **0.006** |
|  | *MT2* | 1.14 | 0.60 | 13.33 | 0.64 |  | 0.94 |
|  | *Gpr50* | 0.96 | 0.05 | 15.44 | 0.56 |  | 0.68 |
| Melatonin receptor gene in the liver | *MT1* | 3.84 | 1.92 | 17.35 | 0.74 |  | 0.002 |
|  | *MT2* | 1.81 | 0.95 | 17.97 | 0.61 |  | 0.03 |
|  | *Gpr50* | 1.75 | 1.29 | 18.41 | 0.62 |  | 0.003 |
| Melatonin receptor gene in the ovary | *MT1* | 0.90 | 0.09 | 9.13 | 0.78 |  | 0.02 |
|  | *MT2* | 1.58 | 0.95 | 17.97 | 0.61 |  | 0.03 |
|  | *Gpr50* | 0.83 | 0.13 | 5.89 | 0.78 |  | 0.02 |
| Melatonin receptor gene in the thymus | *MT1* | 4.44 | 2.64 | 9.48 | 0.39 |  | <0.001 |
|  | *MT2* | 3.18 | 1.69 | 10.72 | 0.25 |  | 0.06 |
|  | *Gpr50* | **2.59** | **1.46** | **10.28** | **0.02** |  | **0.002** |
| Genes involved in general metabolism in the brain | *Sirt1* | **2.10** | **1.51** | **12.45** | **0.15** |  | **<0.001** |
|  | *Fgf21* | **2.22** | **1.62** | **11.96** | **0.22** |  | **<0.001** |
|  | *PPARα* | 1.00 | 0.14 | 19.34 | 0.30 |  | 0.024 |
| Genes involved in general metabolism in the hypothalamus | *Sirt1* | 0.95 | 0.03 | 23.24 | 0.78 |  | 0.001 |
|  | *Fgf21* | 1.21 | 0.09 | 8.36 | 0.92 |  | 0.04 |
|  | *PPARα* | 0.99 | 0.05 | 13.19 | 0.71 |  | 0.003 |
| Genes involved in general metabolism in the liver | *Sirt1* | **2.01** | **1.29** | **15.65** | **0.002** |  | **<0.001** |
|  | *Fgf21* | **1.93** | **1.33** | **14.18** | **0.005** |  | **0.01** |
|  | *PPARα* | 1.85 | 0.91 | 12.91 | 0.4 |  | 0.04 |
| Genes involved in general metabolism in the ovary | *Sirt1* | 0.89 | 0.17 | 21.02 | <0.001 |  | 0.51 |
|  | *Fgf21* | 1.03 | 0.10 | 4.24 | 0.61 |  | 0.75 |
|  | *PPARα* | 1.43 | 0.45 | 10.95 | 0.53 |  | 0.005 |
| Genes involved in general metabolism in the thymus | *Sirt1* | **1.32** | **0.39** | **12.31** | **0.09** |  | **0.004** |
|  | *Fgf21* | 1.29 | 0.45 | 15.82 | 0.22 |  | 0.22 |
|  | *PPARα* | **1.25** | **0.78** | **15.36** | **0.24** |  | **0.004** |

**Note:** Mesor, a rhythm-adjusted mean; Amplitude, a measure of half the extent of predictable variation within one cycle; Acrophase (h), a measure of the time of overall high values recurring in each cycle. There was a statistical significance when both *P* < 0.3 and *P*-value < 0.5 (in bold).
